# Determining the biocontrol capacities of *Trichoderma* spp. originating from Turkey on *Fusarium culmorum* by transcriptional and antagonistic analyses

**DOI:** 10.3389/ffunb.2023.1278525

**Published:** 2023-11-13

**Authors:** Özlem Sefer, Esma Özsoy, Emre Yörük, Evrim Özkale

**Affiliations:** ^1^ Department of Molecular Biology and Genetics, Faculty of Arts and Sciences, Istanbul Yeni Yuzyil University, Istanbul, Türkiye; ^2^ Graduate School of Science and Engineering, Programme of Molecular Biology and Genetics, Yıldız Technical University, Istanbul, Türkiye; ^3^ Institute of Graduate Studies in Sciences, Program of Molecular Biology and Genetics, Istanbul University, Istanbul, Türkiye; ^4^ Department of Biology, Faculty of Science and Letters, Manisa Celal Bayar University, Manisa, Türkiye

**Keywords:** biocontrol agent, *Fusarium culmorum*, Fusarium head blight (FHB), gene expression analysis, *Trichoderma* spp., root rot (RR)

## Abstract

In this study aiming to investigate potential fungal biocontrol agents for *Fusarium culmorum*, several isolates of *Trichoderma* spp. were evaluated for their antagonistic effects by means of transcriptional analyses. At first, 21 monosporic *Trichoderma* spp. isolates were obtained from natural wood debris and wood area soils in Manisa, Turkey. *Trichoderma* spp. Isolates were identified as belonging to four different species (*T. atroviride, T. harzianum, T. koningii*, and *T. brevicompactum*) by *tef1-α* sequencing. Then, the linear growth rate (LGR) of each species was calculated and determined to be in a range between 13.22 ± 0.71 mm/day (*T. atroviride* TR2) and 25.06 ± 1.45 mm/day (*T. harzianum* K30). Inter-simple sequence repeat (ISSR) genotyping validated the *tef1-α* sequencing results by presenting two sub-clusters in the dendrogram. We determined the genetically most similar (TR1 & TR2; 97.77%) and dissimilar (K9 & K17; 40.40%) individuals belonging to the same and different species, respectively. Dual sandwich culture tests (which are useful for antagonism studies) revealed that *T. harzianum* K21 (the least suppressive) and *T. brevicompactum* K26 (the most suppressive) isolates suppressed *F. culmorum* with growth rates of 3% and 46%, respectively. Expressions of genes previously associated with mycoparasitism-plant protection-secondary metabolism (*nag1*, *tgf-1*, and *tmk-1*) were tested by quantitative real-time polymerase chain reaction (qRT-PCR) in both those isolates. While there were no significant differences (p>0.05) in expression that were present in the K21 isolate, those three genes were upregulated with fold change values of 2.69 ± 0.26 (p<0.001), 2.23 ± 0.16 (p<0.001), and 5.38 ± 2.01 (p<0.05) in K26, meaning that the presence of significant alteration in the physiological processes of the fungus. Also, its mycoparasitism potential was tested on *Triticum aestivum* L. cv Basribey *in planta*, which was infected with the *F. culmorum* FcUK99 strain. Results of the trials, including specific plant growth parameters (weight or length of plantlets), confirmed the mycoparasitic potential of the isolate. It can be concluded that (i) *nag1*, *tgf-1*, and *tmk-1* genes could be approved as reliable markers for evaluation of BCA capacities of *Trichoderma* spp. and (ii) the *T. brevicompactum* K26 strain can be suggested as a promising candidate for combating in *F. culmorum* diseases following the necessary procedures to ensure it is non-hazardous and safe.

## 1 Introduction

*Trichoderma* is a fungal genus with more than 340 members, which are mainly found in soil and plants (Sariah et al., 2005; Amin and Razdan, 2010; Strauch et al., 2011). *Trichoderma* spp. is of great importance among filamentous fungi in terms of adapting to different ecological conditions and lifestyles and providing benefits to plant nutrition and immune defence. *Trichoderma* spp., which is saprophytic, contributes to recycling in nature by breaking down dead plant/organic residues and supporting essential nutrients for plant growth (Zeilinger et al., 2016; Singh et al., 2019). Thus, increasing the yield of the plant also prevents plant diseases caused by pathogens by attaching to plant roots and inhibiting the growth (Amin and Razdan, 2010; Mukherjee et al., 2012). Some fungal plant diseases could be controlled by this manner using *Trichoderma* instead of synthetic pesticides. With all these beneficial features and abilities, *Trichoderma* species can be mostly and widely used for biocontrol (Rojo et al., 2007; Zapparata et al., 2021; Yörük et al., 2022).

Microbial biocontrol agents have been preferred in recent years for the fight against the growth of pathogenic microorganisms (Mohamed and Haggag, 2006; Rojo et al., 2007; Zapparata et al., 2021; Yörük et al., 2022). In this context, *Trichoderma* spp. has been found suitable in controlling phytopathogenic fungi such as *Fusarium* sp., *Rhizoctonia* sp., and *Sclerotium* sp. (Elad et al., 1980; Matarese et al., 2012; Abbas et al., 2017; Rai et al., 2019). The most common biocontrol fungi are *T. harzanium*, *T. afroharzanium*, *T. viride*, *T. virens*, and *T. atroviride* (Mohamed and Haggag, 2006; Dubey et al., 2007; Amin and Razdan, 2010; Strauch et al., 2011). As a result, *Trichoderma* species provide a good opportunity to determine a useful strategy for both human/animal health and protecting agricultural products in an eco-friendly manner. In addition, some *Trichoderma* species are also known to show potential for industrial uses such as biodegradation of cellulose and biofuel production (Reithner et al., 2011; Druzhinina and Kubicek, 2017).

In literature, the valuable scientific studies and data on the active or secondary metabolites and volatile compounds produced by *Trichoderma* species are abundant in the study of the gene/gene clusters responsible for this metabolite production; its related activity is rare. Therefore, the molecular basis for the opportunistic success of *Trichoderma* is not yet fully understood, and access to detailed genetic characterization of genes encoding proteins of key importance to biological control strategies in *Trichoderma* spp. isolates is still limited (Küçük and Kivanc, 2005; Küçük et al., 2007; Reithner et al., 2011; Singh et al., 2019; Abbas et al., 2022; Gazdík et al., 2023). There is still limited knowledge of the linkage between genetic characteristics and biocontrol capacities of *Trichoderma* spp. isolates worldwide. Because of this gap in knowledge, studies on gene pathways related to mycoparasitism will gain growing momentum in coming years. Performing expression analyses in different *Trichoderma* spp. strains showing control activity under experimental/environmental conditions will also contribute to the determination of different genome features in terms of functional adaptation.

In recent years, *Trichoderma* sp. has been used as an effective biological control agent in the fight against Fusarium had blight (FHB) and root rot (RR) diseases caused by *Fusarium* spp (Matarese et al., 2012; Zapparata et al., 2021; Yörük et al., 2022; Gazdík et al., 2023; Risoli et al., 2023). FHB and RR diseases have been seen in plenty of plant species and have been often caused by *F. graminearum* and *F. culmorum* species worldwide (Matny, 2015; Yörük and Yli-Mattila, 2019; Mielniczuk and Skwaryło-Bednarz, 2020). These diseases inhibit plant growth, lead to yield loss, and harm the overall health of plants. Not only plant health but also animal and human health are threatened by FHB and RR epidemics. Besides, mycotoxins such as deoxynivalenol and zearalenone are accumulated on small grain cereals and their related products (Goswami and Kistler, 2004; Desjardins and Proctor, 2007; Matny, 2015; Miedaner et al., 2017; Yörük and Yli-Mattila, 2019; Mielniczuk and Skwaryło-Bednarz, 2020). *Trichoderma* species could act by different mechanisms in the control of FHB and RR disease. These mechanisms could be grouped into antagonism, competition, root protection, and stimulation of the plant immune system (Amin and Razdan, 2010; Hermosa et al., 2012; Matarese et al., 2012). In order to use the beneficial effects of *Trichoderma* species, factors such as selection of the appropriate *Trichoderma* species, application dosage, and timing of application must be considered. Also, detailed morphological, transcriptomic, and metabolomic analyses of *Trichoderma* species are needed to create an effective defence strategy.

Morphological and molecular identification of *Trichoderma* species, phylogenetic analysis, determination of mycoparasitic effects, observation of the effects of volatile compound production (on phytopathogenic fungus), detection of the presence of genes related to mycoparasitism and secondary metabolite production, and investigation of the expression levels of these target genes provide important information in terms of their potential use as biocontrol agents (BCA). Obtaining this information would provide an advantage in the use of *Trichoderma* spp. with its antagonistic effects, such as genetically engineered BCAs producing effective metabolites against phytopathogens. However, these positive effects should be confirmed by different morphological, physiological, and genetic methods. This way, within the scope of the current study, genetic identification was performed at the species level after the detailed morphological characterization of *Trichoderma* spp. isolates originating from Turkey. The presence and the expression of genes related to secondary metabolites production by these isolates and their effects as a biocontrol agent on *F. culmorum*, an important plant pathogen, were investigated. Thus, for the first time, identification, detailed genetic characterization, biocontrol potential, and comparative gene expression analysis in *Trichoderma* spp. from Turkey were carried out.

## 2 Materials and methods

### 2.1 Characterization of *Trichoderma* spp. isolates and *in vitro* growth assays

*Trichoderma* spp. isolates, which were previously obtained from natural wood debris and wood area soils from Manisa, Turkey, were used in this study (**Table 1**). Prior to *in vitro* cultivation assays, morphological observations, including branching type, phialide length, and conidia shape, were carried out following the protocols provided by Hoyos-Carvajal et al., 2009. Protocols provided by Irzykowska et al. (2013) and Yörük (2018) for solid and liquid cultures were used with slight modifications. After checking morphological characteristics carefully, each isolate was cultured on potato dextrose agar (PDA) plates for 7 days at 26°C ± 2°C for linear growth rate determination. Carboxymethylcellulose (CMC) medium, inducer medium for spore production, was used in disease severity determination analysis. Up to seven slices obtained from PDA cultures were added to CMC medium, and isolates were incubated at 23°C ± 2°C by shaking 150 rpm.

**Table 1:** *Trichoderma* isolates used in this study.

Code	Species	Accession no	Similarity (%)
TR1	*T. atroviride*	OR487466	99%
TR2	*T. atroviride*	OR487467	93%
TR6	*T. atroviride*	OR487468	99%
TR8	*T. atroviride*	OR487469	99%
TR9	*T. atroviride*	OR487470	99%
TR37	*T. harzianum*	OR487473	98%
TR38	*T. harzianum*	OR487474	99%
TR42	*T. harzianum*	OR487475	99%
TR43	*T. harzianum*	OR487476	99%
TR46	*T. harzianum*	OR487477	99%
TR47	*T. harzianum*	OR487478	99%
K9	*T. harzianum*	OR487479	99%
K17	*T. atroviride*	OR487471	99%
K19	*T. koningii*	OR487485	99%
K20	*T. harzianum*	OR487480	99%
K21	*T. harzianum*	OR487481	99%
K22	*T. atroviride*	OR487472	99%
K24	*T. harzianum*	OR487482	99%
K26	*T. brevicompactum*	OR487486	91%
K27	*T. harzianum*	OR487483	99%
K30	*T. harzianum*	OR487484	99%

For the analysis of the linear growth rates of the 21 isolates obtained, the growth diameters (on PDA media) were measured) on days 4 and 7. The mean values as mm/day were recorded and statistical analyses were performed using descriptive statistics, Tukey’s post-test, and one-way analysis of variance (ANOVA) by Graphpad Prism 9.0. (Dotmatics, U.S.A.).

### 2.2 *tef1-α* gene sequencing-based identification of *Trichoderma* spp

In species-specific identification of *Trichoderma* spp. isolates, gDNAs were isolated from single-spore isolates. SDS-based gDNA isolation was performed from 7-day-old cultures as reported by Niu et al. (2008) and Yörük et al. (2016). Fresh mycelium of 50 mg was homogenized using a microtube homogenizer (Merck-BeadBugTM, Germany) accompanied with 2 mg glass beads by vortexing samples at 3.000 rpm for 30s twice. The homogenized samples were then subjected to digestion, ligation, washing, and elution steps in gDNA isolation. The obtained gDNA molecules were analyzed qualitatively by 1% agarose gel electrophoresis (70 V for 45 min), and quantitatively by spectrophotometer (Thermo, U.S.A.) by measuring the absorbance values at 260 and 280 nm wavelengths for being used in PCR reaction. Quantitative analysis was used to adjust amounts of gDNA for PCRs.

The *tef1-α* gene, which is widely used for species-level diagnosis of fungi, due to it being highly conserved among fungal species, was selected for species-specific identification (Cai and Druzhinina, 2021; Xue et al., 2021; please see https://trichoderma.info/). The final concentrations of the components of PCRs were as follows: 50 ng gDNA, 1X PCR buffer, 2.5 mM MgCl_2_, 0.2 mM dNTP mixture, 10 pmol each primer (O’Donnell et al., 1998), and 0.04 U/μL *Taq* DNA polymerase (Episozyme, Turkey). Following pre-denaturation at 94 °C for 5 min in a thermal cycler, PCR was completed with 35 cycles and a final extension (5 minutes at 72 °C). A total of 35 cycles were performed at 94 °C for 30 s, 57 °C for 30 s, and 72 °C for 45 s. The presence of PCR bands was visualized by 1% agarose gel electrophoresis in the presence of a UV transilluminator at a 70 V constant current for 1 hour. PCR products were purified following the protocol (of the commercial kit) recommended by the manufacturer (Macherey-Nagel, Germany). Sanger sequencing process was performed using a BigDyeTM Terminator kit (Thermo, USA) with DNA sequencing system (Abi Prism-Thermo, U.S.A.). The sequencing reaction was carried out in a reaction volume of 20µL, including a 20ng PCR template, 3 pmol of each primer, and 1X Reaction Buffer by following manufacturer recommendation protocols. The chromatograms were visualized and analyzed with Chromas Pro software (Technelysium, Australia). The sequences were compared for the sequence similarities in the NCBI database by BlastN analysis. E value < 0.05, bit score >50, and query coverage >90% sequences were considered scientifically significant (Altschul et al., 1990). Multiple alignment of the obtained *TEF1-α* sequences was performed with ClustalW online software (please see https://www.genome.jp/tools-bin/clustalw). Aligned sequences were subjected to pairwise distance and Neighbor-Joining topology analysis via Mega 11.0 software and the Bootstrap support value was calculated for the phylogenetic tree with at least 1000 replicates in order to eliminate potential technical errors in phylogenetic analysis (Tamura et al., 2021). Principal component analysis for *TEF1*-α was carried out using R/R-Studio software. ‘.aln’ files obtained from multiple alignments were converted into ‘.phy’ files and then used in PCA analysis by use of R with ‘readxl’, ‘ape’, ‘phangorn’, and ‘factoextra’ packages.

### 2.3 Phylogenetic analysis based on Inter simple sequence repeat markers

Genotyping of *Trichoderma* spp. isolates were performed based on ISSR markers. These markers are particularly useful in genotyping and population genetic studies because they can reveal genetic diversity and relationships among individuals or isolates intra- or inter-specific levels. In total, 10 primers containing di-, tri-, and tetra-nucleotide motifs were used (**Table 2**). In ISSR-PCR, the components were combined in a final concentration consisting of 100 ng gDNA, 1X buffer, 3 mM MgCl_2_, 0.4 mM dNTP mixture, 10 pmol primer, 0.04 U/μL *Taq* DNA polymerase enzyme. The PCR reaction was performed in two-step loops to ensure strong binding of primers to the gDNA template. Following pre-denaturation at 94 °C for 5 min, PCR was performed in two steps. In the first stage, 7 cycles were cycled at 94°C for 1 min, 40°C for 1 min, and 72°C for 3 min. The next 35 cycles were performed at 94 °C for 1 min, 48 °C for 1 min, and 72 °C for 2 min. The final elongation step was completed at 72 °C for 10 min. PCR products were run on 2% agarose gel electrophoresis and visualized under a UV transilluminator. Genetic diversity was analyzed according to the Nei and Li coefficient (Nei and Li, 1979) by scoring the presence of bands with ‘1’ and absence with ‘0’ and using MVSP 3.2.1 (multi-variate statistical package-Kovach). 2D principal component analysis (PCA) graphics and dendrograms were also produced by MVSP software. Polymorphism informative content (PIC) and resolution power (RP) values were also evaluated according to previous publications (Roldan-Ruiz et al., 2000; Prevost and Wilkinson, 1999; Alkan et al., 2019).

**Table 2:** ISSR primers and related data in this study.

Primer	5’-3’ Sequence	Band no	PIC	RIP
UBC866	(ctc)6	13	0.49	12
UBC855	*(ac)8yt*	9	0.48	7.7
UBC867	*(ggc)6*	7	0.493	6.2
UBC872	*(gata)4*	3	0.327	1.2
UBC813	*(ct)8t*	11	0.482	13
UBC825	*(ac)8t*	10	0.489	9.4
AYS6	*(gagg)3gg*	7	0.471	5.3
AYS8	*(gttc)3gt*	9	0.491	10
AYS10	*(cgtg)3cg*	13	0.495	12
UBC828	*(ac)8t*	11	0.499	9.9
**Mean**			0.47	8.67

### 2.4 Mycoparasitic potential of *Trichoderma* spp. on *F. culmorum*

In the study, the *F. culmorum* FcUK99 reference strain was used to demonstrate the mycoparasitic effects and BCA capacity of *Trichoderma* spp. isolates on it. The mycoparasitic effect and inhibition potential of both *Trichoderma* and FcUK99 isolates [the FcUK99 genome project was made, and this isolate has been the subject of many different morphological, physiological, and chemotype analyses and genetic studies (Urban et al., 2016)] were analyzed by subculturing 5-day-old cultures. The inhibition caused by *Trichoderma* species producing volatile compounds was determined by sandwich dual culture technique as reported by Yörük et al. (2022). Sandwich cultures were established in PDA by using a 0.25 cm^2^ agar plug of FcUK99 and *Trichoderma* isolates placed onto the PDA at the same time and conditions as a single culture. For this purpose, sandwich cultures were incubated for 5 days 26°C ± 2°C. The suppression observed in *F. culmorum* was evaluated according to common formula % inhibition = [(mycelial diameter control - mycelial diameter experiment)/mycelial diameter control] x 100 and the isolates were characterized according to the following value ranges (Popiel et al., 2008; Błaszczyk et al., 2017):

- Very active, > 19 mm suppression zone
- Effective, 13-19 mm suppression zone
- Partially active, 10-12.99 mm suppression zone
- No effect, <10 mm suppression zone

### 2.5 Expression analysis of mycoparasitism-related genes in *Trichoderma* spp.

As target genes, *nag1* (N-acetylglucosaminidase) and *tgf-1* (histone acetyltransferase), previously found to be associated with secondary metabolism and mycoparasitism, and *tmk1* (mitogen-activated protein kinase), which is associated with plant protection-secondary metabolism production (Brunner et al., 2003; Reithner et al., 2007; Gómez-Rodriguez et al., 2018), were selected to be used in qRT-PCR assays due to their potential for being a marker gene for mycoparasitism. Total RNA isolation from day 7 mycelium of *Trichoderma* spp. Mono and sandwich dual cultures was performed using Trizol-based monophasic compound (due to universal usage of this agent and its non-tissue/sample specificity). Mycelium (50-200 mg of wet weight) was homogenized with 1 mL of monophasic compound in a digestion device. The nucleoprotein complex was separated with chloroform, and total RNA molecules were precipitated with isoamyl alcohol and solubilized with RNase-free distilled water. After DNAseI treatment (Zymo Research, U.S.A.), Qualitative and quantitative analyses of total RNA were performed by 1% agarose gel electrophoresis and spectrophotometric measurements. From the quantified total RNAs, cDNA translation was performed using a commercial kit (Takara, Japan). cDNA translation followed the protocol recommended by the kit.

Differences in gene expression levels in dual cultures were investigated by qRT-PCR. The α-actin gene encodes a structural cytoskeletal protein and is known to be highly stable. This gene was used as a housekeeping gene, while genes detected by standard PCR were used as target genes. The qRT-PCR primers were designed with Primer3 software by accessing the accessions in the NCBI database (**Supplementary File 1**). qRT-PCR assays were performed based on “SYBR Green I” fluorophore. The reaction was performed in a total volume of 12µL. qR-TPCR Components 50 ng cDNA, 5 pmol forward and reverse primers, and1X SYBR Green Mix (Episozyme, Turkey) were combined in a qRT-PCR 96-wellplate. Samples were kept at 95°C for 2 minutes for pre-denaturation. Replication was performed in 45 cycles of 10 seconds at 95°C, 15 seconds at 60°C, and 20 seconds at 72°C. This was followed by a waiting phase of 30 seconds at 40°C and a melting curve temperature scan. The results were analyzed after three biological and three technical replicates. Standard plots were performed in 4 logarithmic phases at 1/4 dilution ratios. Gene expression profiles will be interpreted according to 2^-ΔΔCT^ normalization values (Livak and Schmittgen, 2001).

### 2.6 *In planta* assays

Two genotypes showing different biocontrol activity for *Trichoderma* spp. as a high suppressor (K26) and low suppressor (K21) for *F. culmorum* FcUK99 strains were selected for in planta tests for evaluating their BCA potential on *F. culmorum*. A common wheat cultivar, *Triticum aestivum* L. cv. Basribey, was used as a plant host. The inoculum source was obtained from *F. culmorum* FcUK99 strain grown on carboxymethylcellulose (CMC) medium at 23 ± 2°C by shaking cultures of 50 mL with 150 rpm rotary shaker speed for 5 days. 1x10^6^ macroconidium (filtered by 2X gauze) was added to the autoclaved soil with the ratio of 5:95/W:V/macroconidium:soil (Moya-Elizondo and Jacobsen, 2016; Moradi et al., 2017). After three days of inoculation, wheat seeds (surface sterilized as reported by Gargouri-Kammoun et al., 2009) and 1x10^7^ spores of *Trichoderma* spp. (grown on potato dextrose broth [PDB] for 5 days at 23 ± 2°C by 150 rpm; including 20 µL Tween20) were incubated for 2 hours 23 ± 2°C by orbital shaker, and then seeds were left to be dried out in laminar flow. After 3 days of soil inoculation with *F. culmorum*, wheat seeds (treated with *Trichoderma* spp.) were planted onto plastic pots of 13 cm diameter and left for plant growth at 16/8 light and dark conditions at 26°C with 1500 lux for 3 weeks. Fresh weight (FW for gram), dry weight (DW for gram), and seedling length (SL for cm) of plantlets were recorded after 3 weeks. Statistical analysis was carried out as reported before using Graphpad Prism 9.0.

### 2.7 Correlation and component assays

2D and 3D analysis were carried out for evaluating different technical protocol sets in a single way. For this purpose, firstly LGR values and mycoparasitism test (BCA) data were co-evaluated by PCA test by R/R-Studio using ‘readxl’, ‘devtools’, and ‘ggbiplot’ packages. Variation values for the dimensions and plots were produced by R. Similarly, by using R/R-Studio, each distinct technical procedure (LGR, BCA, gene expressions, DW, FW, and SL data) was co-evaluated in a single way for K21 and K26.

## 3 Results

### 3.1 Morphological analysis in *Trichoderma* spp.

**Figure 1:**
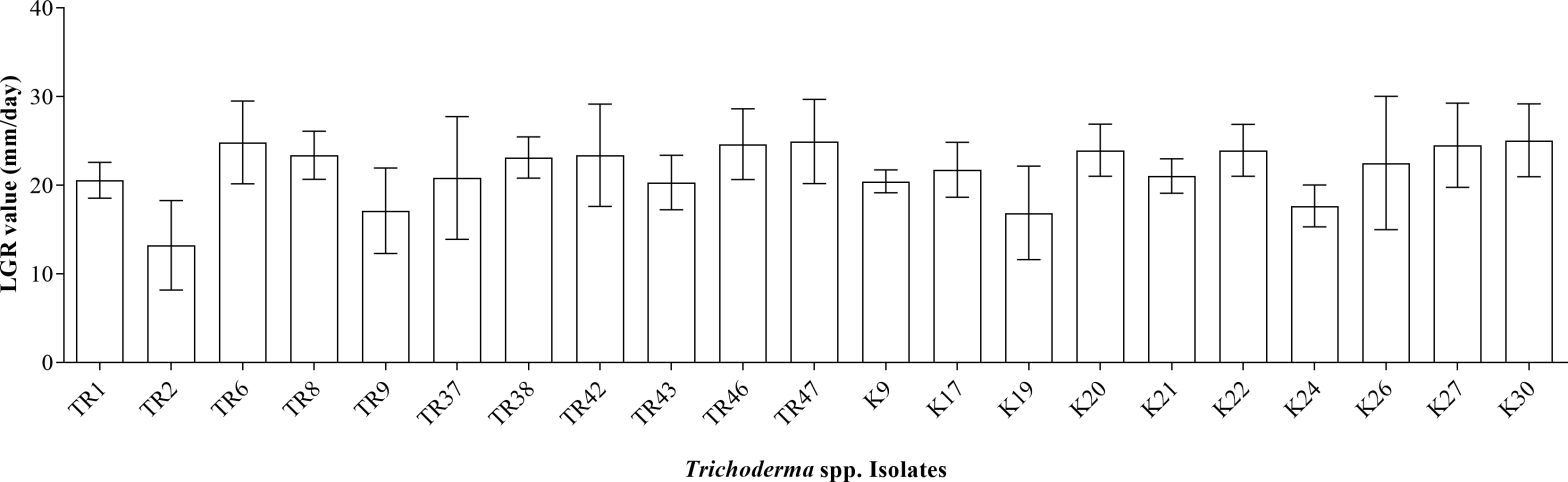
LGR values for 21 *Trichoderma* spp. isolates.

A total of 21 isolates of *Trichoderma* sp. were obtained by single spore isolation protocol from infected plant/soil sources taken from different regions. Four different species were detected via morphological identification criteria. The growth of the isolates (on PDA) was measured on petri on days 4 and 7, and LGRs (mm/day) were calculated. The highest linear growth rate was measured at 25.06 ± 1.45mm/day in the K30 isolate, while the lowest linear growth rate was calculated at 13.22 ± 1.78mm/day in the TR2 isolate (**Figure 1**).

### 3.2 *TEF1-α* sequencing *Trichoderma* spp. isolates

**Figure 2:**
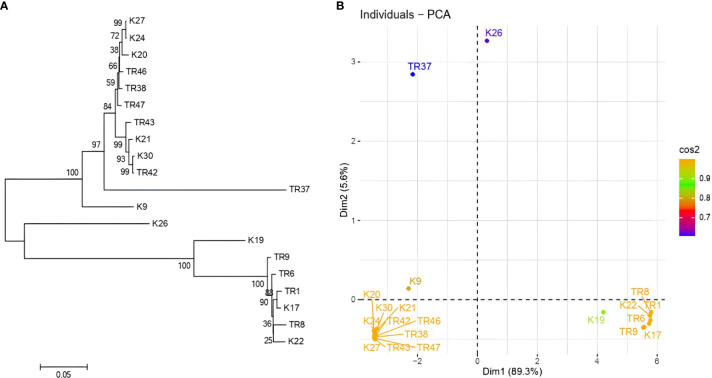
**(A)** NJ topology dendrogram from MEGA 11. Software, and **(B)** PCA graphic from R for *tef1-α* sequencing results in *Trichoderma* spp.

gDNA was successfully isolated from all 21 isolates. *TEF1-α* was amplified ~0.8 kb in all isolates (**Supplementary File 2**), PCR products were purified from agarose gels and then sequenced. After BLASTN analysis, E values were recorded with the range from %91 to %99, respectively (**Table 1**). With the concordance of morphological characteristics of the isolates, 12 isolates belonged to *T. harzianum*, 7 isolates were characterized as *T. atroviride*, 1 isolate as *T. koningii*, and 1 isolate as *T. brevicompactum*. NJ topology analysis easily distinguished all isolates according to their origin of the species (**Figure 2A**). Similarly, PCA assays revealed two major groups with PCA1 and PCA2 scores of percentages of 89.2% and 5.6%, respectively (**Figure 2B**).

### 3.3 Phylogenetic analysis based on ISSR markers

**Figure 3:**
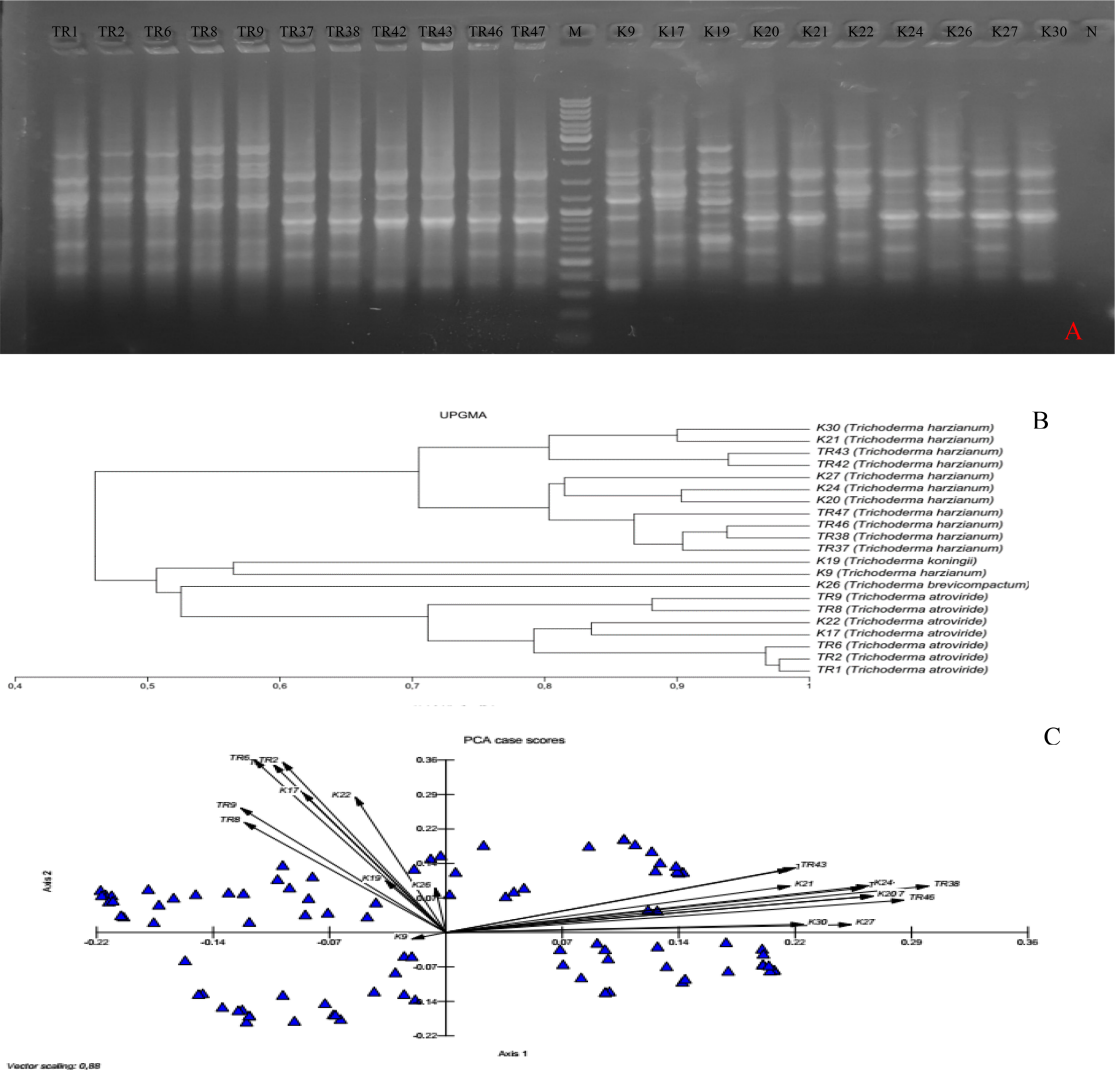
**(A)** UBC828 agarose gel profiling, **(B)** UPGMA dendrogram, **(C)** and PCA plot data obtained from MVSP 3.21 software.

In total, 10 ISSR primers were used in the ISSR PCRs, and all of them yielded PCR products (**Table 2**). It was observed that 93 ISSR bands with 10 ISSR primers were generated in 21 *Trichoderma* isolates, and 88 bands were recorded as polymorphic (94.62%). The maximum number of ISSR bands (13 bands) was observed with the UBC866 and AYS10 primers while the minimum number of bands (3 bands) was recorded with the UBC872 primer. **Figure 3A** presents USSR profiling in the UBC828 primer. According to the ISSR analysis, a similarity matrix revealed that maximum similarity was present between TR1 and TR2 *T. atroviride* isolates with 97% similarity. Minimum similarity was detected as 30.6% between TR46 (*T. harzianum*) and TR8 (*T. atroviride*). The dendrogram (**Figure 3B**) and PCA biplot (**Figure 3C**) showed phylogenetic branching through two main branches. The members of *T. harzianum* and *T. atroviride* species showed co-clustering. Among the 21 isolates, 2 isolates with different species (*T. koningii* and *T. brevicompactum*) and showed a distant branching to *T. atroviride* and *T. harzianum* species.

### 3.4 BCA potential of *Trichoderma* spp. isolates on *F. culmorum*

**Figure 4:**
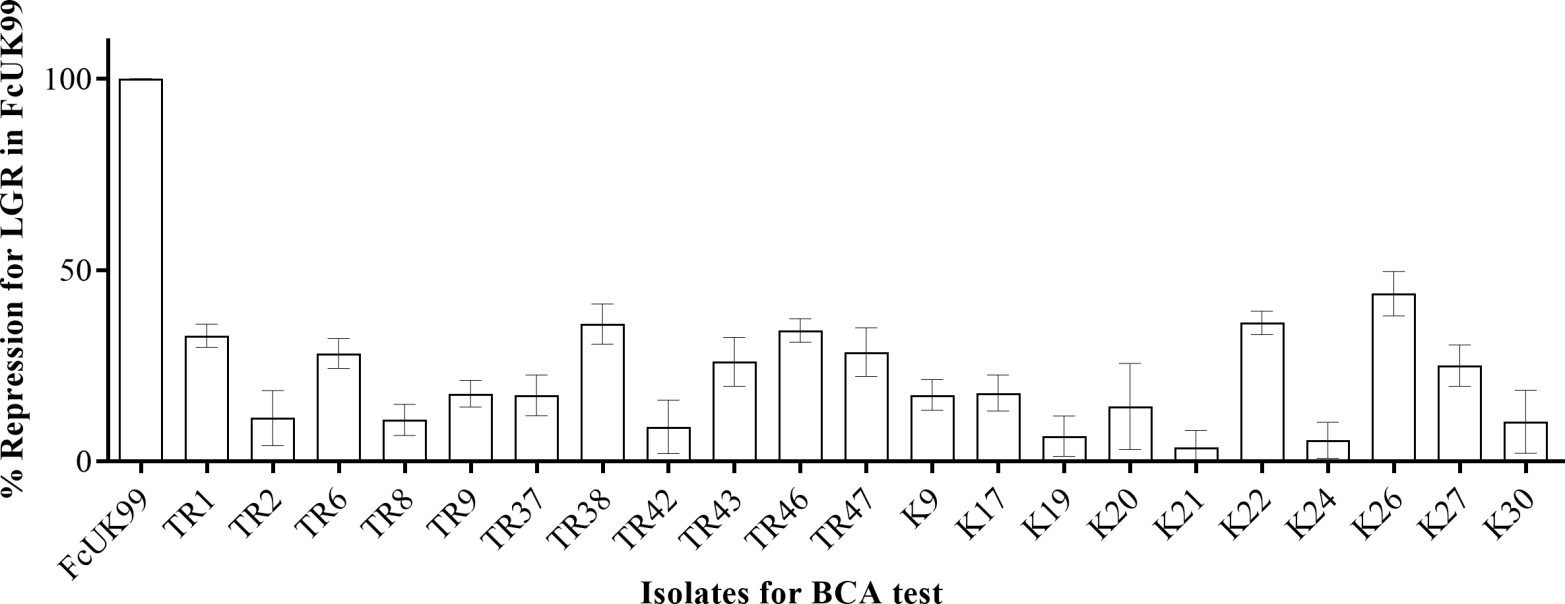
BCA potential of each *Trichoderma* isolates by % suppression values.

LGR for FcUK99 treated with *Trichoderma* spp. isolates via sandwich cultures were recorded at the days 3, 4, and 5 of incubation. According to the measurement results obtained, among 21 different *Trichoderma* spp. isolates, the highest mycoparasitic effect on FcUK99 was found to be isolate K26 with 43% suppression, while the lowest mycoparasitic effect was observed in isolate K21 with 3% suppression (**Figure 4**). The overall mycoparasitic effect of all isolates was measured mean percentages of 20.77 ± 2.49. K21 and K26 isolates were selected for further molecular analysis.

### 3.5 qRT-PCR analysis of mycoparasitic related genes in *Trichoderma* spp.

**Figure 5:**
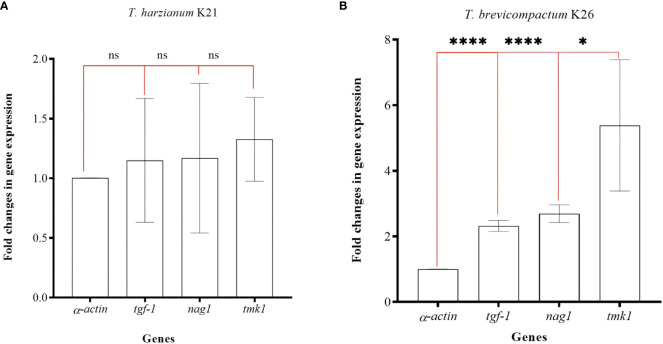
Fold changes in gene expression values for three genes in K21 **(A)** and K26 **(B)** isolates. ‘ns’ means no significant changes, ‘*’ means p>0.05, and ‘****’ means p<0.001.

In the study, K21 and K26 isolates were subjected to gene expression analysis. qRT-PCRs were used in expression analysis of the *tgf-1, nag1*, and *tmk1* genes. Target gene expressions were normalized according to the *α-actin* gene. Total RNAs with high quality (Δ;_260/280 _= 1.9-2.0) and quantity (1-2 µg/µl) were obtained. The fold changes in expression of the *tgf-1, nag1*, and *tmk1* genes were calculated as 1.15 ± 0.51, 1.16 ± 0.62, and 1.32 ± 0.35, respectively. No significant differences (p>0,05, ns) were present in the K21 isolate (**Figure 5A**). However, fold changes in gene expression levels of the *tgf-1, nag1*, and *tmk1* genes in the K26 isolate were determined as 2.69 ± 0.26 (p<0.001, ****), 2.23 ± 0.16 (p<0.001, ****), and 5.38 ± 2.01 (p<0.05, *) respectively (**Figure 5B**). The expression of *tgf-1, nag1*, and *tmk1* genes was found to be significantly altered in K26.

### 3.6 *In planta* analysis

**Figure 6:**
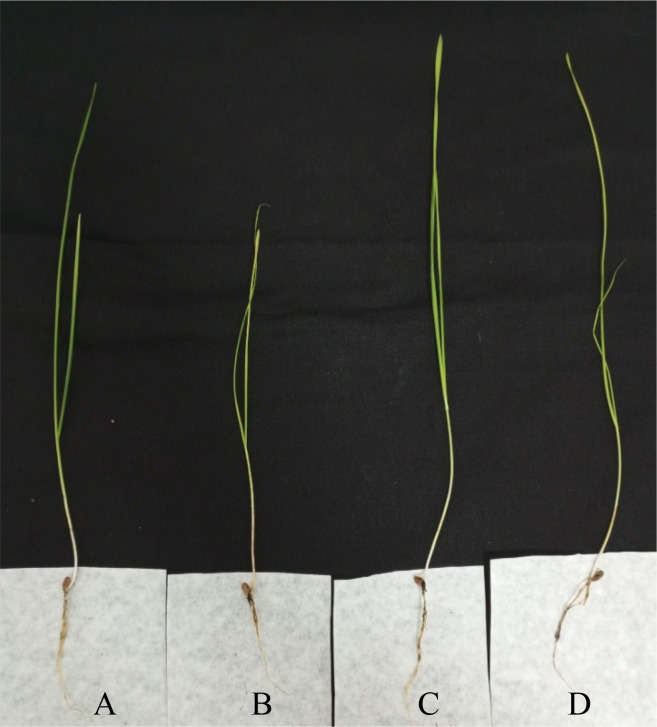
3-week-old plantlet of *T. aestivum* L. cv. Basribey. **(A)** Control plant (non-infected and non-BCA treated), **(B)** positive control (only phytopathogen infected plant), **(C)** experiment set for K21 (FcUK99 infected and K21), and **(D)** experiment set for K26 (FcUK99 infected and K26 treated plant).

After conducting the connection platform for phytopathogen, BCA, and plant host, physiological changes in plantlets were recorded. This way, the mycoparastism potential of selected *Trichoderma* isolates was tested on *Triticum aestivum* L. cv Basribey infected with *F. culmorum* FcUK99 strain. FW values were recorded as 0.18 ± 0.01 gr, 0.06 ± 0.008 gr (p<0.001, ****), 0.15 ± 0.02 gr (p>0.05, ns), and 0.15 ± 0.02 gr (p>0.05, ns) for the control plant (non-infected and non-BCA treated), positive control (only phytopathogen infected plant), experiment set for K21 (FcUK99 infected and K21), and experiment set for K26 (FcUK99 infected and K26 treated plant), respectively (**Figure 5**). In comparison to the control set, both K21 and K26 reduced the adverse effects of FcUK99 on wheat. DW values were calculated as 0.018 ± 0.001 gr, 0.009 ± 0.0009 gr (p<0.01, ***), 0.013 ± 0.001 gr (p>0.05, ns), and 0.015 ± 0.001 gr (p>0.05, ns) for the control plant (non-infected and non-BCA treated), positive control (only phytopathogen infected plant), experiment set for K21 (FcUK99 infected and K21), and experiment set for K26 (FcUK99 infected and K26 treated plant), respectively (**Figure 5**). Like DW results, both *Trichoderma* isolates reduced the adverse effects of FcUK99 on wheat. SL values were recorded as 25.58 ± 1.06 cm, 20.12 ± 2.07 cm (p<0.05, *), 24.62 ± 1.99 cm (p>0.05, ns), and 26.78 ± 0.52 cm (p>0.05, ns) for the control plant (non-infected and non-BCA treated), positive control (only phytopathogen infected plant), experiment set for K21 (FcUK99 infected and K21), and experiment set for K26 (FcUK99 infected and K26 treated plant), respectively (**Figures 6A–D**). Like FW and DW data, *Trichoderma* isolates reduced the potential growth of pathogen in wheat. The decreased level of disruption in *F. culmorum-infected* plantlets was clearly related to *Trichoderma* treatment.

### 3.7 Correlation and component analysis

**Figure 7:**
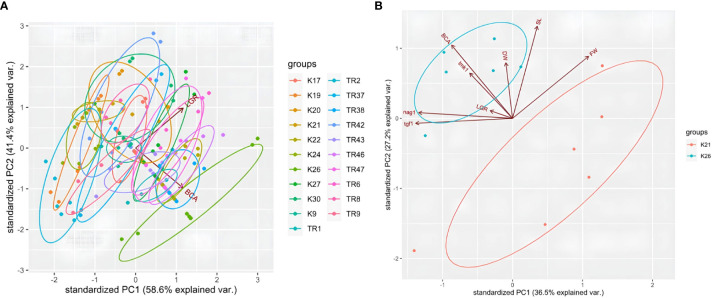
PCA plot for LGR and BCA analysis in 21 *Trichoderma* spp. isolates **(A)**, and plot for K21 and K26 with LGR, BCA, tgf1, nag1, tmk1, DW, FW, and SL data **(B)**.

For LGR and BCA analysis, a PCA test was carried out using R. According to the PCA plot, data obtained for each *Trichoderma* isolate co-clustered in regard with species origin. Especially, *T. atroviride* isolates were distributed within the same ellipses for LGR data (**Figure 7**). Even if *T. koningii* K19 isolates were co-clustered in *T. harzanium* isolates and it showed similar pattern for LGR and BCA data, a clear distinction was present for *T. brevicompactum* K26 isolates for PCA biplot (**Figure 7A**). Percentages for axis 1 and axis 2 were 58.6% and 41.4% (showing a high level of variation for two different sets of test data), respectively. A PCA graphic was also obtained by way of R for a detailed comparison of tests carried out for the BCA capacity of K21 and K26 isolates (**Figure 7B**). A great degree of variation (with PC1 of 36.5% and PC2 of 27.2%) was detected for the characterization of K21 and K26 isolates by PCA test. Nearly every experimental procedure but LGR clearly distinguished between the two contrasting *Trichoderma* genotypes.

## 4 Discussion

The stress induced by treating plants with synthetic antifungal compounds promoted the resistance development in phytopathogenic fungi severely. The development of resistance in FHB and RR pathogens to common fungicides is estimated to be a highly severe problem in the next years (Chung et al., 2008; Yin et al., 2009; Zheng et al., 2015; Perlin et al., 2017). More effective disease management strategies (besides phytopathogen identification, crop rotations, novel compound usage, etc.) are needed to combat FHB and RR. In recent years, plant-derived secondary metabolites (PDSM) have started being used for *in vitro* tests. However, the amount of the PDSM needed for *in vitro* tests have been reported to be relatively higher in comparison to synthetic antifungal compounds (Elgat et al., 2020; Kong et al., 2022; Albayrak et al., 2023). Moreover, the production of PDSM (essentially essential oil derivatives) by biotechnological companies and/or obtaining these compounds could be non-cost-effective, laborious, and time-consuming. Also, *in planta* tests for PDSM effectiveness on plant protection are missing from the literature for nearly all publications. Thus, other strategies such as BCA usage could be more useful in FHB and RR disease management and control.

There is an extensive history of the use of *Trichoderma* spp. as a BCA against *Fusarium oxysporum* and therefore *T. harzianum* is the most widely known biocontrol species against phytopathogenic fungi (Mohamed and Haggag, 2006; Amin and Razdan, 2010; Yang et al., 2011; Hermosa et al., 2012; Rawat et al., 2013). *T. asperellum*, *T. hamatum*, and their products (culture filtrates or enzymes) have also been reported to be useful for use as biocontrol agents (Solanki et al., 2011; Kumar et al., 2012; Rai et al., 2019). However, in recent studies *T. atroviride* and other *Trichoderma* sp. strains have been focused on for controlling FHB, RR, and other *Fusarium* diseases (Matarese et al., 2012; Gómez-Rodriguez et al., 2018; Bouanaka et al., 2021; Zapparata et al., 2021; Özkale et al., 2023). In this study, members of four different *Trichoderma* species have been evaluated in terms of their mycoparasitisc capacities due to expressional analyses. In comparison to the results of previous studies, the BCA capacities of *Trichoderma* species used in this study against *F. culmorum* are of the acceptably effective and highly effective levels (Popiel et al., 2008; Błaszczyk et al., 2017). In particular, data obtained from LGR, BCA, and in planta tests revealed that the *T. brevicompactum* K26 isolate is found to be a strong suppressor of the growth of *F. culmorum* with BCA, LGR, DW, FW, and SL values of 43.99 ± 2.31, 22.5 ± 2.65, 0.016 ± 0.01, 0.15 ± 0.022, and 26.98 ± 0.47 respectively. The determination of the disease (FHB or RR) severity changes in *Trichoderma* spp.-treated plantlets could be investigated in further studies. Similarly, determination of *Fusarium* spp. infection levels in plant tissues after *Trichoderma* spp. application can also be investigated by qRT-PCR. The use of a qRT-PCR-based strategy for evaluating the potential BCA capacity of *T. brevicompactum* for *Fusarium*-infected plantlets is still missing from the literature. Besides, testing this activity of the isolate against the other *Fusarium* species related to FHB and RR diseases, such as *F. graminearum*, *F. pseudograminearum*, and *F. avenaceum*, in further studies would be very fruitful.

Phylogenetic analysis obtained from *TEF1-α* and ISSR fingerprinting revealed that two main sub-divisions, containing *T. harzianum* and *T. atroviride* isolates separately, were present in dendrograms and PCA plots. Moreover, both *tef1*-α and ISSR dendograms and *TEF1-α* and ISSR PCA plots presented *T. brevicompactum* and *T. koningii* isolates at the edge and extremes of the clusters. Similar results were obtained from ISSR and ITS sequencing data in *Trichoderma harzianum* and *Trichoderma viride* isolates from Delhi-India (Kumar and Sharma, 2011). However, they reported that genetic polymorphism analysis that they used did not establish a relationship between *Trichoderma* spp. isolates and their antagonism potential. In this study, similar patterns were detected between genetic diversity values and *in vitro* growth and the BCA capacity of the isolates. Moreover, contrast genotypes, K21 and K26, presented different patterns for their genetic similarity, mycoparasitism potential, and transcriptional activity for mycoparasitism-related genes.

Previous studies have not revealed a clear relationship between the mycoparasitism capacity, *in vitro* growth potential, mycoparasitism gene expressions, and plant protective effects of *Trichoderma* isolates (Ghutukade et al., 2015; Mazrou et al., 2020). In particular, correlation between mycoparasitism-related gene expression and physiological and genetic characteristics of *Trichoderma* isolates is missing from the literature. In addition, PIC and RP values showed that ISSR primers selected in this study presented high level of polymorphism and detailed genetic characterization for *Trichoderma* isolates. Mean PIC (0.47) and RP (8.67) values showed high levels of polymorphism in comparison to previous polymorphism studies (Jingade et al., 2018; Alkan et al., 2019; Aulakh et al., 2021; Yörük et al., 2022). Findings obtained from this study revealed that UBC866, UBC867, and AYS10 primers could provide good opportunity to distinguish *Trichoderma* isolates via ISSR fingerprinting assays.

Several similar studies on the relatedness between *Trichoderma* spp. genes and its mycoparasitic potential (Brunner et al., 2003; Reithner et al., 2007; Mukherjee et al., 2012; Sharma et al., 2013; Gómez-Rodriguez et al., 2018; Abbas et al., 2022) are found in literature. However, limited research on BCA-related genes and their potential use in characterizing *Trichoderma* isolates for their biocontrol capacities have been carried out. Three different genes and their relationships with mycoparasitism have already been confirmed previously (Brunner et al., 2003; Reithner et al., 2007; Gómez-Rodriguez et al., 2018), and they were used in gene expression analyses in this study. Each gene was significantly upregulated in a sandwich dual culture of K26 isolate in comparison to monoculturing of the strain. On the other hand, there were no significant changes for those three genes in K21 isolate. Our findings revealed that there were two contrast expression profiles in two contrast *Trichoderma* genotypes. K26, (the most. suppressive of *Fc*), showed significantly increased expressions of mycoparasitism-related genes when it was treated with *F. culmorum* in sandwich cultures. But no alteration in the expressions was quantified for K21 (relatively low level suppressive of *Fc*) in dual culture conditions. These findings concluded that (i) the genes selected in this study can be used as reliable marker genes for discriminating *Trichoderma* individuals for their biocontrol capacities, and (ii) changes in the expression of these genes may also be correlated with other physiological, genetic, and mycoparasitic characteristics of *Trichoderma* isolates. Therefore, further studies should be carried out by whole exome sequencing and/or ChiP-Seq experiments to provide more detailed information on *Trichoderma* isolates and their different mycoparasitic capacities.

## Data availability statement

The data presented in the study are deposited in the GenBank repository, accession numbers OR487466-OR487486 with release date 26 September, 2024.

## Author contributions

ÖS: Conceptualization, Data curation, Methodology, Writing – original draft. EsÖ: Methodology, Writing – original draft, Investigation. EY: Methodology, Writing – original draft, Data curation, Formal Analysis. EvÖ: Methodology, Resources, Supervision, Writing – review & editing.
