# Supplementary figures and images for: Determining the biocontrol capacities of Trichoderma spp. originating from Turkey on Fusarium culmorum by transcriptional and antagonistic analyses
Source: Front Fungal Biol. 2023 Nov 13;4:1278525. doi: 10.3389/ffunb.2023.1278525 (PMC10679392; doi:10.3389/ffunb.2023.1278525)

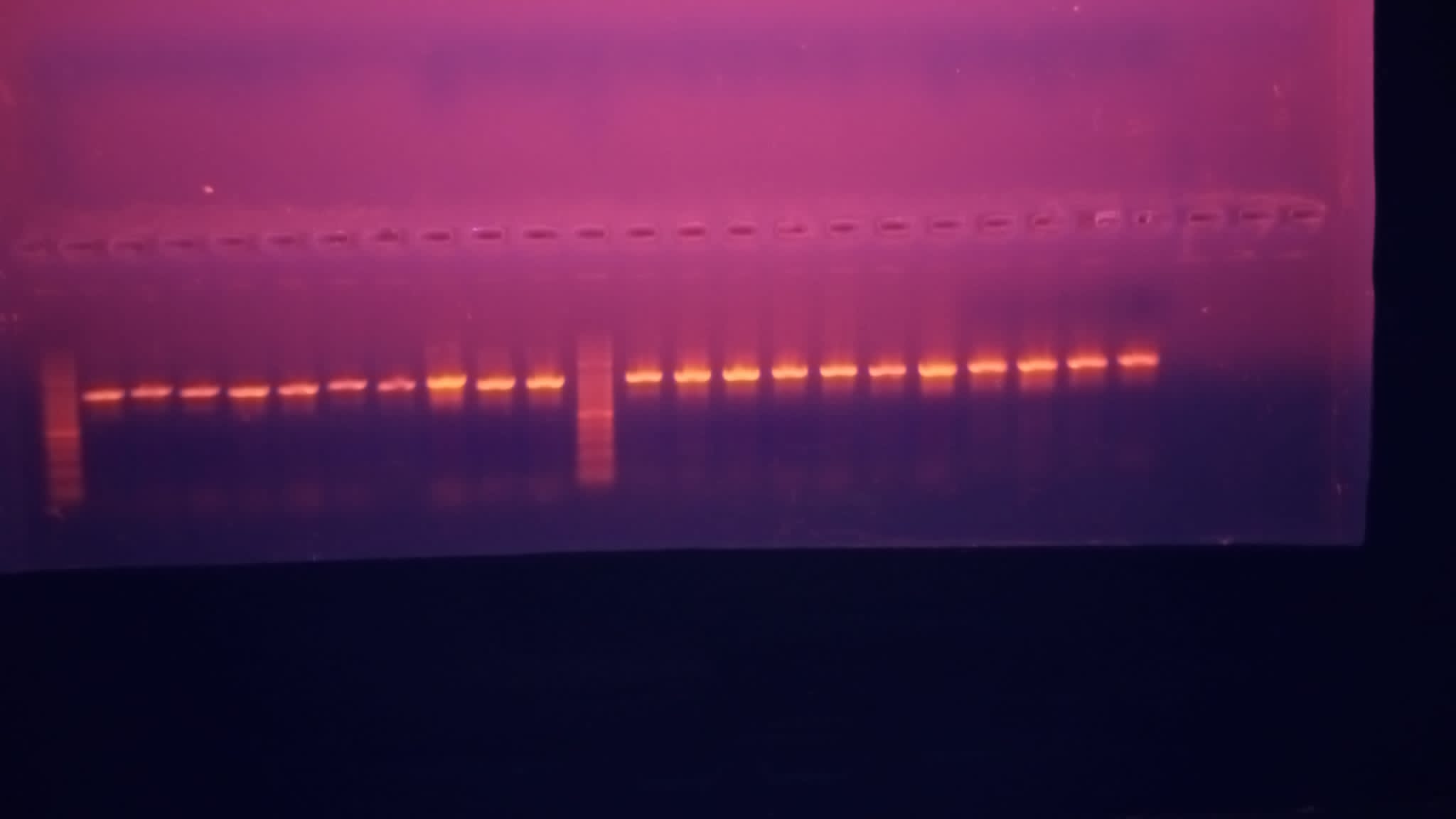

Supplement: Supplementary file 1 [file Image_1.jpeg]
