# Supplementary material for: Determining the biocontrol capacities of Trichoderma spp. originating from Turkey on Fusarium culmorum by transcriptional and antagonistic analyses
Source: Front Fungal Biol. 2023 Nov 13;4:1278525. doi: 10.3389/ffunb.2023.1278525 (PMC10679392; doi:10.3389/ffunb.2023.1278525)
Supplement: Supplementary file 2 [file Table_1.docx]

| **Primer set** | **Forward sequence (5ˈ-3ˈ)** | **Reverse sequence (5ˈ-3ˈ)** | **Target gene** | **Band size in bp** |
| --- | --- | --- | --- | --- |
| Act fw / Act Rv | TCACCGAGGCCCCCATCAACC | CGACCGGAAGCGTACAGGGACAGA | *α-actin* | 127 |
| Tanag1f / Tanag1r | ACTTTGTGCCATGGAAGCTG | GCACTGATGTTGACCTGTCC | *nag1* | 193 |
| Tshaf / Tshar | CGACGCAGATGAACGAGATG | AGAGTTACAGCCAAGCCCAT | *tgf-1.* | 250 |
| Tatmk1f / Tatmk1r | CTCCTTCAATGTCAGCGAGC | TCGTGGTTGAAATAGCGCAG | *tmk1* | 192 |
